# An Antidote for Hydrophobia

**Published:** 1884-06

**Authors:** 


### AN ANTIDOTE FOR HYDROPHOBIA.

THE celebrated French chemist M. Louis Pasteur claims to have discovered a complete antidote for hydrophobia. In an interview with a Paris *Figaro* correspondent he is reported as saying:

"Cauterization of the wound immediately after the bite, as is well-known, has been more or less effective, but from to-day anybody bitten by a mad dog has only to present himself at the laboratory of the Ecole Normale and by inoculation I will make him completely insusceptible to the effects of hydrophobia, even if bitten subsequently by any number of mad dogs.

"I have been devoting the last four years to this subject. I found out, in the first place, that the *virus rabique* loses its intensity by transmission to certain animals and increases its intensity by transmission to other animals. With the rabbit, for instance, the *virus rabique* increases; with the monkey it decreases. My method was as follows: I took the virus direct from the brain of a dog that had died from acute hydrophobia. With this virus I inoculated a monkey. The monkey died.

"Then with the virus—already weakened in intensity—taken from this monkey I inoculated a second monkey. Then with the virus taken from the second monkey I inoculated a third monkey, and so on until I obtained a virus so weak as to be almost harmless. Then with this almost harmless virus I inoculated a rabbit, the virus being at once increased in intensity.

"Then with the virus from the first rabbit I inoculated a second rabbit, and there was another increase in the intensity of the virus. Then with the virus of the second rabbit I inoculated a third rabbit, then a fourth, until the virus had regained its maximum intensity.

Thus I obtained virus of different degrees of power. I then took a dog and inoculated him, first with the weakest virus from the rabbit, then with the virus from the second rabbit, and finally with the rabbit virus of maximum intensity. After a few days more I inoculated the dog with virus taken directly from the brain of a dog that had just died of acute madness. The dog upon which I had experimented proved completely insusceptible to hydrophobia. The experiment was frequently repeated, always with the same successful result.

"But my discovery does not end here. I took two dogs and inoculated them both with virus taken directly from a dog that had just died of acute hydrophobia. I let one of my two dogs thus inoculated alone, and he went mad and died of acute hydrophobia. I subjected the second dog to my treatment, giving him the three rabbit inoculations, beginning with the weakest and ending with the strongest. The second dog was completely cured, or rather became completely insusceptible to hydrophobia."

[A more recent statement attributed to M. Pasteur is that dogs can be made proof against hydrophobia by inoculating them with the virus carefully selected and obtained by a certain process; but it is understood that for the present at least, the idea of protecting men against hydrophobia by inoculation is entirely out of the question.—Ed. Journal of Health.]

